# Rad52 deficiency decreases development of lung squamous cell carcinomas by enhancing immuno-surveillance

**DOI:** 10.18632/oncotarget.16371

**Published:** 2017-03-18

**Authors:** Rachel Lieberman, Jing Pan, Qi Zhang, Ming You

**Affiliations:** ^1^ Department of Pharmacology & Toxicology, Medical College of Wisconsin, Milwaukee, WI, USA; ^2^ Cancer Center, Medical College of Wisconsin, Milwaukee, WI, USA

**Keywords:** Rad52, DDR (DNA damage response), lung cancer, SCC (squamous cell carcinoma), HR (homologous recombination)

## Abstract

RAD52 is involved in homologous recombination and DNA repair. This study focuses on lung cancer progression and how the DNA repair gene, Rad52, enables tumor cells to have sufficient genome integrity, i.e., the ability to repair lethal DNA damage, to avoid cell death. In this report, we analyze the phenotypic differences between wild type and Rad52^−/−^ in inhibition of tumor phenotypes including cell growth, viability, cytolysis, and immune profiling. We demonstrated that loss of Rad52 not only increases the death of cells undergoing carcinogen-induced transformation *in vivo*, but that Rad52 loss also augments *in vivo* antitumor activity through an enhanced capacity for direct killing of LLC tumor cells by stimulated Rad52^−/−^ NK and CD8^+^ T cells. We hypothesize that upon DNA damage, wild type cells attempt to repair DNA lesions, but those cells that survive will continue to divide with damage and a high likelihood of progressing to malignancy. Loss of Rad52, however, appears to increase genomic instability beyond a manageable threshold, acceding the damaged cells to death before they are able to become tumor cells. Our results suggest a key role for the complex interplay between the DNA damage response and host immunity in determining risk for Squamous Cell Lung Carcinoma.

## INTRODUCTION

More people die of lung cancer than of breast, prostate, and colon cancers combined, with an estimated incidence of over 200,000 cases in 2016 [1]. While therapeutic advances are occurring, such as molecular targeted therapies against activating mutations of epidermal growth factor receptor (EGFR) and anaplastic lymphoma kinase (ALK) translocations, these have only helped sub-populations of patients at best, and often only for brief periods during their disease course [2]. Clearly, novel therapeutic strategies for both early and late stage lung cancer are needed.

Like most cancers, lung cancer is believed to arise from the accumulation of mutations in DNA that eventually disrupt the ability of a cell to both manage interactions with its environment and control its rate of proliferation [3]. However, while lung cancer likely arises due to acquisiton of somatic mutations, contemporary evidence has begun to show that genetic variants also play a role in cancer progression and prognosis in patients [4, 5].

RAD52, a protein important for DNA double-strand break repair in homologous recombination, was first associated with NSCLC risk through a Genome-wide association study conducted in Europeans which linked the 12p13.33 locus containing *RAD52* with squamous cell lung cancer risk [6, 7]. Previously, RAD51 and OGG1 have been shown to repair DNA damage and increase cellular resistance to oxidative stress, and RAD52 mediates RAD51 function in homologous recombinational repair (HRR) in both the yeast, *Saccharomyces cerevisiae*, and in mammalian cells of mice and humans [8, 9]. However, while the DNA damage response (DDR) has historically been suggested to represent a barrier against tumorigenesis by preventing the uncontrolled proliferation of cells with genomic instability or harmful mutations, recent studies have uncovered novel links of the DDR to immune signaling pathways.

Lung cancer progression is a multi-step mechanism encompassing chronic inflammation due to an imbalance in cytokine secretions and inflammatory responses which permit cancer cells to evade surveillance and elimination by the host immune system, ultimately favoring the malignant transformation of normal epithelial cells [10, 11]. In this study, we evaluated the contribution of Rad52 activity on lung tumor growth in C57BL/6 mice. Our results demonstrate that Rad52 depletion increased cell death, decreased myeloid cell frequency, and augmented the incidence and activity of CD8+ T cells and NK effectors that ultimately led to reduced tumor growth. Our data provides support for the notion of RAD52 as a potential oncogene, implicating a major role for the combined processes of recombinational repair and host immunity in determining risk for Squamous Cell Lung Carcinoma (LUSC).

## RESULTS

### Effect of Rad52 depletion on mouse lung SCC development

**Figure 1:**
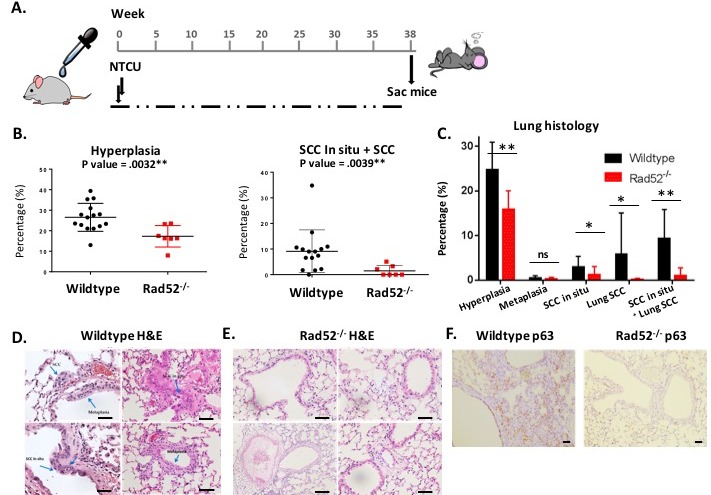
Loss of Rad52 decreases carcinogenesis in mice treated with NTCU **A**. A design for evaluating Rad52^−/−^ against lung carcinogenesis in C57Bl/6 mice. Lung tumors are induced twice weekly by NTCU painting. **B**.-**C**. Histologic results of 38 weeks of NTCU painting comparing wild type (*n* = 13) to Rad52^−/−^ mice (*n* = 8). **D**.-**E**. Representative images of H&E staining of mouse lungs after treatment (magnification 10x). Blue arrows point to SCC pearls (top left panel), SCC *in situ* (top left arrow in bottom left panel) and hyperplastic bronchioles (bottom right arrow in bottom left panel) and **F**. Representative images of p63 (squamous cell carcinoma marker) after treatment (magnification 5x). *p < 0.05, **p < 0.005 and ***p < 0.001 were considered to be statistically significant.

In our model of lung squamous cell carcinoma (SCC), we induced lung SCCs using the carcinogen N-nitroso-tris-chloroethylurea (NTCU) as described in Materials and Methods [12]. Because C57BL6/J mice are more resistant to carcinogen-induced lung tumors than most other mouse strains, we extended the period of topical NTCU treatment from 24 weeks to 38 [13, 14] (Figure 1A). Rad52 knockout mice did not exhibit enhanced signs of ill health, nor have significant loss of body weight following treatment with NCTU compared to wild type.

NTCU induces premalignant lesions that progress to frank lung SCC, resembling the stepwise progression observed during the development of lung SCC in humans [15]. Histologic assessment of lung tissue after 38 weeks of bi-weekly NTCU treatment revealed significant differences in tumor cell growth between wild type and Rad52^−/−^ strains (Figure 1B-1C). Lung sections were stained with H&E to evaluate lung architecture, which clearly indicated dense staining of hyperplastic bronchial lobes and keratin pearl arrangement indicative of squamous cell carcinoma in wild type mice (Figure 1D) [12]. Under light microscopy, normal bronchi are seen as a single layer of bronchial epithelial cells (Figure 1E). While SCCs typically lack somatic oncogene-activating mutations, they exhibit frequent overexpression of the p53-related transcription factor p63 [16]. Lung tissues in Rad52^−/−^ mice showed very little p63 staining, which is consistent with the reduced development of SCC observed histologically (Figure 1F). These observations suggest that depletion of Rad52 decreases both hyperplasia and SCC.

### *In vivo* micronucleus assay detects genome instability in Rad52^−/−^ mice

**Figure 2:**
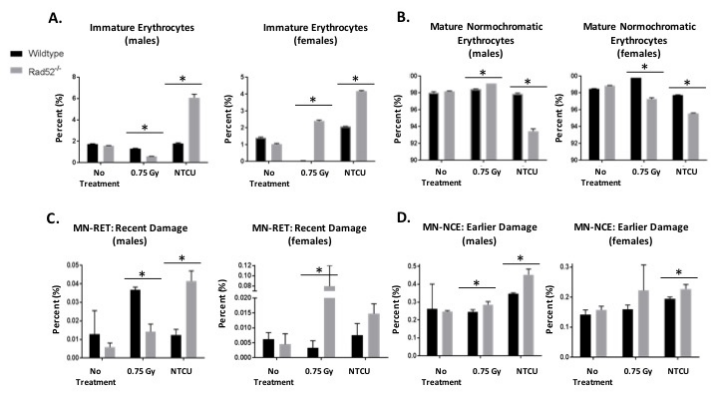
*In vivo* micronucleus assay detects genome instability in Rad52−/− mice Cells which are genomically unstable or mice that have been treated with a genotoxin have a higher frequency of micronucleus formation. Mouse blood samples are collected into liquid heparin solution and fixed in cold methanol. Samples are prepared and incubated in buffer containing FITC-conjugated CD71 antibody and RNase. Samples are washed and resuspended in buffer plus PI and analyzed by collecting 200,000 events by flow cytometry. Micronuclei are PI-positive, and they can be differentially identified in NCEs or RETs by co-staining with CD71. Mice are either not treated, treated with 0.75 Gray irradiation, or treated with 38 weeks of NTCU painting. **A**.-**B**. First, percentages of immature to mature erythrocytes were analyzed. **C**.-**D**. Then, DNA damage was measured as indicated by incidence of micronuclei. Micronucleated RETs are indicative of recent damage, whereas micronucleated NCEs are indicative of damage caused > 72 h earlier. P-value and significance calculated to only compare wild type v. knockout in each individual treatment group and quadrant. Multiple testing adjustments were performed so that the threshold would be less than the Bonferroni correction using *p* < 0.05 as threshold. *p < 0.0167 was considered to be statistically significant. Wild type mice (*n* = 13); Rad52^−/−^ mice (*n* = 8).

In addition to histologic lung staining, blood samples were collected from each NTCU-treated mouse through retro-orbital bleed upon reaching the endpoint of the experiment (Figure 2). Small amounts of blood were analyzed for the formation of micronuclei (MN), a marker of genomic instability in mouse erythrocytes according to the modified method of Adams and McIntyre [17]. Levels of MN increased significantly in female and male Rad52^−/−^ mice treated with NTCU, and in female Rad52^−/−^ mice exposed to irradiation (Figure 2C-2D). Interestingly, we also observed heightened levels of immature erythrocytes in Rad52^−/−^ mice and decreased levels of mature normochromatic erythrocytes (NCEs) in mice treated with NTCU (Figure 2A-2B). This suggests that upon exposure to cytotoxic treatment, loss of Rad52 induces a level of instability within the erythrocyte progenitor, leading to immature RBCs in the peripheral circulation.

### NTCU treatment in Rad52^−/−^ mice is associated with induction of late apoptosis and necrosis

**Figure 3:**
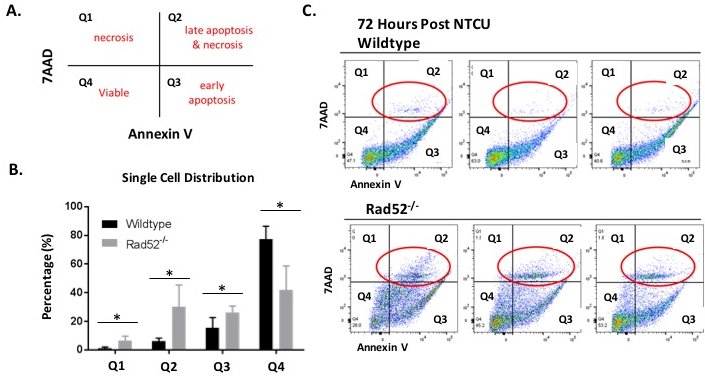
Changes in plasma membrane upon NTCU treatment detected by Annexin V assay Detection of apoptosis and necrosis by concurrent staining with annexin V-APC and 7-AAD. **A**. Live cells (Viable) are both annexin V and 7AAD negative. At early stage of apoptosis (early apoptosis) the cells bind annexin V while still excluding PI. At late stage of apoptosis and early necrosis they bind annexin V and stain brightly with 7AAD. In exclusively necrotic cells, they stain only 7AAD. **B**.-**C**. Wild type mouse lung cells (C. top panel) and Rad52^−/−^ mouse lung cells (C. bottom panel) were treated with NTCU for 72 h, as described in Materials and Methods. Cells were digested with MACS lung digestion kit and subsequently stained with annexin V - APC conjugate and 7AAD and their fluorescence was measured by flow cytometry. P-value and significance calculated to compare wild type v. knockout in each individual quadrant. Multiple testing adjustments were performed so that the threshold would be less than the Bonferroni correction using *p* < 0.05 as threshold. *p < 0.0125 was considered to be statistically significant. Wild-type (*n* = 6); Rad52^−/−^ mice (*n* = 6).

Based on our previous results demonstrating enhanced cell death upon Rad52 depletion *in vitro* and decreased incidence of LUSC in Rad52^−/−^ mice *in vivo*, we wondered whether loss of Rad52 may also sensitize bronchial cells in the murine model to cell death due to excessive genomic insult by NTCU treatment. We used Annexin-V/7-AAD staining of whole lung digests by flow cytometry to investigate the potential negative impact on cell viability of wild type *versus* Rad52 knockout mouse lung cells (Figure 3). Representative Annexin-V/7-ADD dot plots confirm increased late apoptosis and necrosis in Rad52^−/−^ mouse lungs at 72 h post-NTCU treatment (Figure 3B-3C). Annexin-V/7-AAD staining demonstrate an increase in necrotic cells in Rad52−/− mice by the upper left quadrant (Annexin-V-/7-ADD +) and an increase in late apoptotic/necrotic cells in the upper right (annexin-V+/7-AAD+ and annexin-V-/7-AAD+) upon a 72 h treatment with NTCU (Figure 3A).

### Elevated T cell and decreased myeloid cells in lung infiltrates from Rad52^−/−^ mice

**Figure 4:**
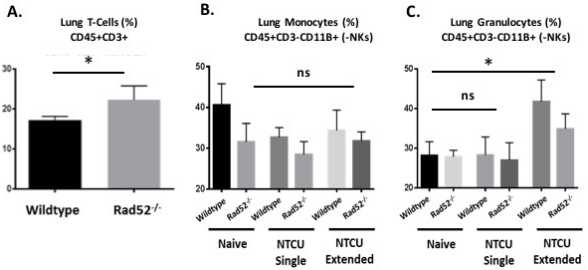
Immune cell populations by flow cytometry from naïve and NTCU-treated lungs Hematopoietic cell lung infiltrates from naïve lung and in response to NTCU challenge. Wild type and Rad52 knockout C57/B6 mice were treated with NTCU or PBS alone. Whole lungs were digested for flow cytometry analysis after only PBS (Naïve, no treatment), 1 dose of NTCU (NTCU Single), or 4 treatments of NTCU (NTCU Extended, 4 doses of NTCU applied 72 hours apart). **A**. Live, untreated cells were selected by forward- and side-scatter, and populations of T cells (CD45+ CD3+), and **B**.-**C**. granulocytic and monocytic myeloid naïve and NTCU-treated cells (CD11b+ Gr-1/Ly-6G^High^ Ly-6C^low^ and CD11b+ Gr-1/ Ly-6G-/^low^ Ly-6C^high^) were quantified. ns, no significance; *p < 0.05, **p < 0.005 and ***p < 0.001 were considered to be statistically significant. Wild-type (*n* = 6); Rad52^−/−^ mice (*n* = 6).

Previously published data have described the induction of the innate immune system in response to DNA damage [18]. We therefore assayed immune cells from whole lung at 0, 3, and 14 days after NTCU treatment (treatment given every 3 days) because this was the point at which differences between wild type and Rad52^−/−^ were observed. We examined populations of T cells (CD3+), and lung monocytes and granulocytes (CD11b+ Gr-1/Ly-6GHigh Ly-6Clow and CD11b+ Gr-1/ Ly-6G-/low Ly-6Chigh) in wild type and Rad52^−/−^ strains with and without NTCU challenge (Figure 4). When comparing populations of naïve T cells from total lung digests, we observed a consistent trend toward increased T cell populations in the knockout mice (Figure 4A). Percentages reached significance, with a P value equal to 0.031. We also observed consistent trends in genetic-dependent differences in CD11b+ Gr-1/ Ly-6G-/low Ly-6Chigh monocytic lung cells in naïve and NTCU-treated mice (Figure 4B) and in CD11b+ Gr-1/Ly-6GHigh Ly-6Clow granulocytic lung cells only upon NTCU treatment (Figure 4C). The observed trends reached a level of significance in comparing levels of granulocytic myeloid cells between genotypes, with wild type mice demonstrating greater increases in granulocytic myeloid cells upon extended NTCU treatment compared to knockout (*p* values = 0.0411 and 0.0218, respectively).

### Sensitization of tumor cells to cell-mediated lysis by Rad52^−/−^ NK and CD8+ T cells

Due to the differences by genotype we observed in immune panels of the lung, we next wanted to assess whether immunologic components, such as cytotoxic CD8+ T cells (CTLs) and NK cells, were involved in eliciting tumor cell lysis.

**Figure 5:**
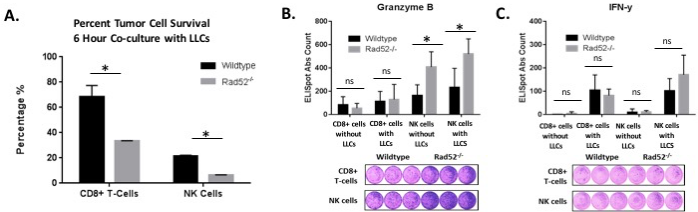
Tumor cell lysis measured by luciferase activity in the surviving target cells Luciferase-expressing Lewis lung carcinoma tumor cells (25,000/well) were plated with and without immune effector cells (750,000/well) at an E: T ratio (Effector to Target) of 30:1. **A**. CD8+ T cells and NK cells were isolated from either wild type or Rad52 knockout mouse spleens, stimulated for 72 h with CD3/CD28 beads with 50 U/ml IL-2 (T-cells) or 1000 U/ml IL-2 (NK cells), and co-cultured with 25,000 luc+ LLC target cells/well. Tumor cell viability was assessed by BLI after 6 h of co-culture (described in Materials and Methods). The % tumor cell survival of Lewis lung cells obtained by the BLI assay is plotted for isolation of both CD8+ T cells and NK cells. Results are represented as mean ± SD of *n* = 3 independent experiments. Decreased LLC survival was observed in LLC target cells when co-cultured with splenocytes depleted of Rad52. The % survival of Lewis Lung Carcinoma (LLC) cells was calculated using the formula: % survival = (mean BLI test signal/mean BLI maximal signal) × 100. In these assays, LLC cell survival is a direct reflection of T cell-mediated lysis and correlates with classical chromium release assays. **B**.-**C**. Levels of granzyme B and IFN-y were calculated using ELISpot kits from R&D Systems. Immune cells were plated either without target cells (CD8+ cells without LLCs and NK cells without LLCs) or with target LLCs at E:T ratios of 1:5 (10,000 effector immune cells: 50,000 target tumor cells). P-value and significance calculated to only compare wild type v. knockout in each individual treatment group. Multiple testing adjustments were performed so that the threshold would be less than the Bonferroni correction using *p* < 0.05 as threshold. *p < 0.0125 was considered to be statistically significant. ns, Not Significant. Wild-type (*n* = 3); Rad52^−/−^ mice (*n* = 3).

Cytotoxicity of target cells is a major function exhibited by CTLs and NK cells. To determine the killing effects of CTLs and NK cells, we isolated T cells and NK cells from naïve mice and stimulated them as described in Materials and Methods. IL-2-stimulated CTLs and NK cells were added separately to wells with LLCs at an E:T ratio of 30:1 and incubated for 6 hours at 37°C. LLC lysates were then obtained and analyzed for luminescence relative light units (RUIs) as described in Materials and Methods. LLCs without added splenocytes were used as a control to account for the maximum RUI to be detected from the plated number of target cells. After 6 h, the wild type CTLs reduced the cell-associated luciferase activity by approximately 30% while knockout CTLs reduced the luciferase activity by 70% (Figure 5A). Likewise, wild type NKs reduced the cell-associated luciferase activity to approximately 25% relative to LLCs cultured in the absence of any NK cells, while NK cells isolated from Rad52 knockout mice reduced the luciferase activity to about 6% of the control BLI consisting of LLCs without co-cultured NK cells (Figure 5A).

The major cytotoxic proteins that enable NK and CTL effector function are the granzymes, perforin, and IFN-y [19–21]. We assessed whether the aforementioned granzyme B (GrB) and interferon-y (IFN-y) secretion correlated with the observed tumor cell lysis observed *in vitro*. As literature suggests, since the GrB ELISpot assay mainly detects stimulated and not spontaneous (constitutive) release of GrB, we first stimulated the isolated splenocytes with recombinant murine IL-2 prior to assay incubation [22]. We found that in Rad52^−/−^ NK cells and CD8^+^ T cells, GrB spots per well were increased by 60% and 12%, respectively, compared to wild type (Figure 5B). Alternatively, in NK cells, IFN-y spots per well were increased 33% but just missed significance with a p-value of 0.054, while CD8+ T cells did not produce any significant difference in secretion due to genotype (Figure 5C). These results may be indicative of low amounts of IFN-y produced, making measurement difficult, or due to the nature of CD8+ T cells being a part of the adaptive immune response. Germline knockout of Rad52 appears to more readily effect NK activity and the innate immune response.

## DISCUSSION

An incomplete understanding exists in the published literature regarding the role of RAD52 and the DNA damage response (DDR) in regulating cell survival during tumorigenesis. Often times, a correlation between expression of members of the DDR and the onset of apoptosis has led to the conclusion that all facets of the DDR are directly involved in defeating tumorigenesis. However, the results of this study suggest that Rad52 activity could occur as part of the tumor cell defense, aiding in malignant progression to enhance tumor cell viability. Genetic instability is indeed necessary for malignant transformation; however, the transformation process requires that an appropriate balance between genomic stability and instability be reached, (i.e., overexpressing specific DNA repair genes like Rad52) in order to generate clones that are genetically stable enough to continue to proliferate, avoid apoptosis and/or necrosis, and to resist cellular stressors. In this case, homologous repair by Rad52 may be allowing tumor cells to retain adequate stability in the face of DNA-damaging agents in order to thrive and progress towards malignancy. We hypothesize that incurred genetic instability upon loss of Rad52 may lead to the observed cell death.

In the present study, we explored tumorigenesis in a Rad52^−/−^ mouse model. Our studies focus on the role Rad52 plays in the progression of pre-malignancy to squamous cell carcinoma of the lung. We discovered that the incidence and progression of both premalignant and fully malignant lesions was decreased in Rad52^−/−^ mice compared to wild type mice. Although this is an inherently interesting phenotype, the question of mechanism remains. Cells face continuous exposure to endogenous and exogenous DNA damage, such as single-strand and double-strand breaks (DSBs), which require unremitting activation of DNA repair pathways [23]. *RAD52* seems to support lung tumor cells in remaining genetically stable enough to proliferate and avoid cell death. Interestingly, upon treating mice with single dose NTCU treatment we observed an increase in the number of cells in the stages of late apoptosis or necrosis in Rad52 knockout mice compared to wild type. This mirrors the role of p53 in managing the balance between cell death and cell survival in maintaining homeostasis through aborting pre-cancerous cells with detected DNA damage instead of restoring their genome. Specifically, the Brash group showed that in the context of skin cancer induced by ultraviolet light, p53-mediated apoptosis eliminates UV-damaged cells, while without functional p53, cells accumulate mutations, become resistant to apoptosis, and are likely to become malignant [24]. Our observations show that upon carcinogen challenge, loss of Rad52 leads to decreased mature erythrocytes and increased genetic instability in murine blood, as well as enhanced cell death in the lungs, suggesting that Rad52-depleted cells may succumb to cell death when faced with such a challenge instead of restoring their genome and progressing toward malignancy. Thus, if fewer cells survive the initial carcinogenic insult, there may be fewer damaged cells able to proceed to tumorigenesis; in essence, the scale may be tipped too strongly toward genomic instability at baseline, causing even relatively minor insults to tip pre-malignant cells into crisis—and ultimately necrotic cell death. As phenotypes in the blood are often systemically representative of other processes, this may be indicative of the enhanced levels of cell death we observed upon depletion of Rad52.

Furthermore, an interplay between the DNA damage response and the immune system is now evident [18, 25, 26]. Genotoxic or replicative stress triggers a DNA damage response that induces cell cycle arrest, DNA repair, or cell death if the damage is too severe. In these circumstances, DNA damage sensors also have the ability to alter immune signaling. One of the most representative links between innate immunity and DDR is the activation of natural killer group 2 (NKG2D) in DNA-damaged cells by ataxia telangiectasia mutation (ATM), which alerts and recruits NK cells at the injured site [27]. In addition, CTLs and NK cells drive immune responses through initiation of apoptosis in the affected cells through the release of cytolytic lysosomal granules [21]. It has been consistently observed in multiple human tumor types that the presence of tumor infiltrating lymphocytes is associated with better patient survival [28]. However, T cells eventually fail to eradicate the tumor in all cancer patients, and cytotoxic CD8^+^ T cells present in the tumor microenvironment of lung cancer patients were actually observed to be hyporesponsive to activation *via* the T cell receptor (TCR) and less effective compared to those exposed to a non-tumor environment [29]. Adding to the problem, NK cell function is also compromised in lung cancer, with NK cells demonstrating a decreased ability to degranulate and produce interferon (IFN)-γ, resulting in decreased cytotoxicity [11, 30, 31].

Increasingly, immunotherapies are coming to the forefront of lung cancer therapy as both monotherapies and combination therapies [2, 32] and our data suggest that the increased cell death found in NTCU-treated Rad52^−/−^ mouse lungs may be due to an altered immune response. Here, we focus on NK cells of the innate immune response and CD8^+^ T cells of the adaptive immune response. Previous reports have demonstrated that proteins involved in genomic instability and the DDR can influence the differentiation and maturation of T cells and other lymphocytes [33]. A recent report found that ATM knockout leads to a T cell maturation defect, resulting in decreased numbers of circulating, mature CD4+ and CD8+ T cells. Interestingly, this group showed that crossing Atm knockout mice with Rad52 knockout mice partially rescued this phenotype, with Atm, Rad52 double knockout mice demonstrating improved mature CD4+ and CD8+ counts relative to Atm knockouts [34]. We found a similar phenomenon at work in our present studies, with a trend toward increased T cell lymphocyte count as a result of Rad52 knockout. This may indicate that Rad52 plays a critical role in determining myeloid *versus* lymphoid lineage commitment in progenitor marrow cells, or that Rad52 itself may represent a checkpoint important in T cell maturation.

Clearly, the combination of increased DNA damage, an enhanced DDR, and a naturally decreased immune response renders lung cancer cells as formidable opponents to treatment. We determined that loss of Rad52 in murine CD8+ T cells and NK cells increases tumor cell cytotoxicity as measured by cell-associated luciferase activity. *Ex vivo* analysis of these cell populations revealed that Rad52 knockout CTLs, as well as NK cells, have enhanced anti-tumor activity compared to wild type. Additionally, while granzyme B and interferon-y are both widely used for measuring tumor-specific responses, ELISpot results suggest that granzyme B is the more specific indicator of CTL and NK cytotoxic ability. These findings are in concert with previous studies which suggest that GrB plays a critical role in controlling tumors *in vivo* as it is able to access the target cell cytosol, where it processes key substrates to initiate cell death [35–39].

In this report, we dissect the phenotypic distinctions between wild type and Rad52^−/−^ in inhibition of tumor phenotypes including cell growth, viability, cytolysis, and immune profiling. Our data suggests that loss of Rad52 not only augments *in vivo* antitumor activity as demonstrated by an enhanced capacity for direct killing of LLC tumor cells by stimulated Rad52^−/−^ NK and CD8^+^ T cells, but that knockout lung cells more readily undergo cell death rather than transformation when exposed to carcinogen. Modulation of immune suppression and activation has been an area of intensive investigation, and our results highlight the complexities that underlie regulation of tumor cytolysis. Effective therapy will require bypassing suppressive factors by countering immune-suppressive environments through methods such as checkpoint blockade or generation of immune-activating environments [40–42]. RAD52 diminution may be more effective if accomplished in the early stages of disease progression, as opposed to later stages and in accordance with other DNA repair inhibitors.

While the data described here provide novel insight into the role of Rad52 and the DNA damage response in both tumorigenesis and the immune response, future work needs to expand upon these concepts. One important avenue would be to determine whether the defect observed in immune cells upon knockout of Rad52 is a cell intrinsic or extrinsic effect. It is possible that loss of Rad52 within the immune cells is not the direct issue and the heightened immune response is actually due to an environmental shift within the host or the tumor microenvironment that alerts the immune system and enhances the immune response. Such a reshaping of the host environment could cause major problems for a developing tumor cell if the resulting environment favors normal cells instead of tumorigenesis. For instance, we showed that loss of Rad52 leads to enhanced cell death. If cells are becoming apoptotic or necrotic early on due to an inherent increase in genomic instability combined with a genotoxic challenge, there is a better chance that these cells will die before they ever start becoming malignant.

To untangle this concept various experiments could be designed. Performing a bone marrow transplantation by engrafting Rad52^−/−^ mouse bone marrow into CD45.1 non-alloreactive wild-type congenic mice could potentially be one method to determine whether Rad52^−/−^ immune cells retain their differential physiology in a wild-type environment or if it is actually the Rad52^−/−^ environment that is responsible for the immune cell defect. A second experiment may involve the Rag-2^−/−^/γC^−/−^ mouse, which combines the Rag-2 knock-out with a null mutation for the common gamma chain receptor [43]. Here, one could employ adoptive transfer experiments in which RAD52 deficient immune cells would be administered to either wild type mice or mice lacking mature T and B cells to detect the presence or absence of the supposed protective factor upon induction of carcinogenesis. Similarly, in determining if the immune system is indeed essential for the observed decrease in tumorigenesis in Rad52 knockout mice, models such as the Nude mouse and the NOD SCID mouse strains would also represent systems for testing immunogenicity [44]. Both mouse models would work in distinct ways and answer different questions regarding immunogenicity in the context of Rad52 activity. For instance, because nude mice lack T cells, but retain NK cell function, the necessity of Rad52 deficient T cells could be determined; a reduction in killing potential would suggest that T cells are a necessary component. Finally, to look at differences in immune infiltrates between wild-type and knockout mice and to determine what exactly is happening immunologically within the tumor, future experiments could be designed to inoculate groups of wild-type and knockout mice in their flanks with wild-type LLC tumor cells. With this, tumor growth rate, immune cell infiltrates and splenic phenotypes at the point of tumor burden could be analyzed for further consideration.

As previously described, our studies suggest that cell death, tumor inhibition, and immunity are enhanced in mouse cells depleted of RAD52. Loss of Rad52 appears to increase genomic instability beyond a manageable threshold, consenting the damaged cells to death before they are able to become tumor cells. Our results suggest a key role for the complex interplay between the DNA damage response and host immunity in determining risk for LUSC. Further investigations into the link between mediators of the DDR and tumorigenesis will shed additional light on this important facet of NSCLC disease progression.

## MATERIALS AND METHODS

### Reagents and animals

NTCU was purchased from Toronto Research Chemicals (Toronto, Canada) and stored at −80°C. Acetone was purchased from Sigma (St. Louis, MO). NTCU was diluted in acetone weekly for treatment and stored for up to one week at 4°C. We acquired the Rad52 transgenic model from The European Mutant Mouse Archive (EMMA, Munchen, Germany) as sperm which were later cryo-recovered by Jackson Laboratories (Bar Harbor, Maine) in the C57B6/J mouse inbred strain. All animal studies were carried out in accordance with IACUC-approved protocols in a pathogen-free AAALAC certified facility.

### NTCU model

Mouse LUSC models induced by NTCU were established as previously reported [12, 45, 46]. All studies on animals were approved by the Medical College of Wisconsin Institutional Animal Care and Use Committee (Milwaukee, Wisconsin). Eight-week-old C57B6/J male and female mice were obtained from Jackson Laboratories. Animals were housed with wood chip bedding in environmentally controlled, clean-air rooms with a 12-hour light-dark cycle and a 50% relative humidity. Drinking water and diet were supplied *ad libitum*. The sub-scapular region of the mice was shaved prior to the first NTCU treatment. Aliquots of NTCU or vehicle (acetone) were applied topically with 0.03 mol/L NTCU in 100-microliter drop, twice a week, with a 3.5-day interval for 38 weeks. During the studies, the health condition of the mice was monitored daily and body weights were measured bi-weekly. Thirty-eight weeks after the initial treatment of NTCU, mice were terminated by CO2 asphyxiation. Toxicity from the carcinogen was manifested as weight loss, superficial skin lesions, and lethargy. If weight loss was greater than 10%, treatment was interrupted and mice were allowed to recover. Missed treatments were added to the end of the study in order to maintain a total delivered dose of 15 μmol or 25 μmol NTCU. Mice were euthanized and lung lobes were either inflated with 10 % neutral buffered formalin (VWR) and paraffin embedded or flash frozen in liquid nitrogen and stored at −80°C for isolation of RNA.

### Scoring histopathology and staining

Lung tissues, which were fixed in 10% buffered formalin overnight and then stored in 70% ethanol, were cut (5 μm each) for future immunohistochemical analysis. All slides (five per mouse) were deparaffinized in xylene and rehydrated in gradient ethanol. Microwave antigen retrieval was carried out by treating the slides for 20 min with citrate buffer (pH 6.0). After blocking in 10% normal goat serum in PBS, the tissue slides were treated with primary antibody and incubated at 4°C overnight. Cell proliferation was assessed using primary monoclonal antibody against p63 (1:400 dilution; Lab Vision Corp.). Negative control slides were processed at the same time. Manual counting of labeled and total cells in high-powered (×400) fields of tumor tissue was conducted.

Unlike lung adenomas/adenocarcinomas, SCCs do not form visible solid nodules on the surface of the lung. Serial tissue sections (5 μm each) were made from formalin-fixed lungs, and one in every 20 sections (approximately 100 μm apart) was stained with hematoxylin and eosin (H&E) and p63 and examined histologically under a light microscope to assess severity of tumor development (invasive SCC, SCC *in situ*, bronchial hyperplasia, metaplasia) as was reported previously [15]. The cross-sectional bronchial cuts were counted on all of the slides. The lesions, including invasive SCC, SCC *in situ*, and bronchial hyperplasia/metaplasia, were scored. The criteria for histopathologic examination and scoring were described previously [15].

Briefly, in hyperplastic lesions (Figure 3D) a single layer of bronchiolar epithelial cells becomes multiple layers. The cells maintain their normal morphology. In metaplastic lesions, the normal columnar epithelium is replaced by flattened squamous epithelium with increased keratin production. In SCC *in situ* lesions, atypical cells (irregular shape, increased nuclear/cytoplasmic ratio) with visible mitosis and loss of orderly differentiation replace the entire thickness of the epithelium, although the bronchial basement membrane remains intact, with no tumor cell invasion into the surrounding stroma. In invasive SCCs, general features of SCC, such as keratin pearls, multiple nuclei, and increasing mitotic index can be seen. The normal architecture of the lung is disrupted. The lung SCC area/lung lobe area ratio was evaluated using NanoZoomer Digital Pathology Virtual Slide Viewer software (Hamamatsu Photonic Co). H&E-stained slides were scanned with the NanoZoomer HT slide scanner (Hamamatsu Photonics France SARL) and virtual slides analyzed and quantified.

### Micronuclei assay

Blood samples were taken from mice treated with NTCU for 38 weeks through retro-orbital bleeds and used for quantifying the formation of micronuclei, markers of genome instability, in mouse erythrocytes according to the modified method of Adams and McIntyre [17]. Briefly, blood samples each containing approximately 25 µl of blood were collected into liquid heparin solution and fixed in cold methanol. Samples were washed, incubated in buffer containing FITC-conjugated anti-transferrin receptor (CD71) antibody and RNase, and stained with Propidium Iodide (PI) before collecting 200,000 events by flow cytometry. Micronuclei are PI-positive and can be differentially identified in mature and immature erythrocytes by co-staining with CD71 (Figure 2). We compared levels of micronuclei in NTCU-treated mice to both naïve mice and mice exposed to a single dose of 0.75 Gray irradiation. Micronucleated reticulocytes (RETs) are indicative of recent damage, whereas micronucleated normochromatic erythrocytes (NCEs) are indicative of damage caused > 72 h earlier [17].

### Isolation of murine lung cells

Mouse lungs were removed from the chest cavity, and a lung single-cell suspension was prepared through proteolytic digestion of the lungs to isolate epithelial, endothelial, and immune cells. Reagents were purchased from Miltenyi Biotec, Inc. (Bergisch Gladbach, Germany) and included the Mouse Lung Dissociation Kit (cat no. 130-095-927). Lungs were digested according to the gentleMACS protocol (Miltenyi Biotec, Bergisch Gladbach, Germany). Briefly, lungs were harvested from each mouse, single lobes were finely minced using scalpels, and the minced tissue was added to a tube containing 2.4 ml of the enzyme mixture for 30 min at 37°C. After digestions, 10 ml of DMEM with 10% FBS was added, each sample passed through a 70-micron filter and treated with ACK cell lysis buffer (Lonza, Walkersville, MD). Single cell suspensions were then held on ice until ready to use.

### Analysis of apoptosis by flow cytometry

Cell death was assayed using Annexin V (BD Biosciences Pharmingen, San Jose, CA) staining. Annexin V binds to the phospholipid phosphatidylserine. As cells undergo apoptosis, phosphatidylserine is translocated from the internal plasma membrane out to the extracellular environment [47]. 7-AAD (BD Biosciences Pharmingen), a dye used for cell viability staining is excluded by intact, viable cells and is found intracellularly in dead or dying cells. Single cell populations were stained with Annexin V and 7-AAD according to the manufacturer's protocols. Briefly, 2×10^5^ to 5×10^5^ stained cells were washed in cold PBS and resuspended in 100 μl of 1X binding buffer (BD Biosciences Pharmingen). Then, 5 μl each of Annexin V-APC and 7-AAD were added to each tube. After gentle vortexing, the cells were incubated at RT in the dark for 15 min. The volume of each tube was brought up to 500 μl with 1X binding buffer.

### Immune panel by flow cytometry

Single-cell suspensions of lung samples were stained with PE-conjugated antibody for CD45; APC E780-conjugated antibody for CD3; APC-conjugated antibodies for CD62L, Ly6G, and NKp46; E450-conjugated antibodies for CD8 and CD11B; FITC-conjugated antibodies for CD4, Ly6C, and CD11C; and PECY7-conjugated antibodies for CD44, CD19, and F4/80 (eBioscience, Immunocell, Singapore; BD Biosciences Pharmingen, NJ). Fc-blocking Ab (Clone 2.4G2; BioLegend, Genomax, Singapore) was used before staining with specific Abs at 4°C. Acquisition of samples was performed on Cyan ADP (Beckman Coulter, CA) or Fortessa (BD Biosciences Pharmingen). Data were analyzed using FlowJo v9.3 software (Tree Star, Ashland, OR).

### Isolation of murine splenocytes

A single-cell suspension was prepared from the spleens of wild type and knockout mice. Spleens were removed from the mice, placed in RPMI medium with 10% FBS, mashed and put through a 70-micron cell strainer. Erythrocytes were depleted using ACK red blood cell (RBC) lysis buffer.

### Immune cell purification and stimulation

Splenic NK cells were isolated using the EasySep Mouse NK Cell Isolation Kit (STEMCELL, Singapore) according to the manufacturer's protocol. Isolated cells were stimulated in culture with 1000 U/ml mouse recombinant IL-2 (Thermo Fisher Scientific, Waltham, MA). CD8+ T cells were isolated using EasySep™ Mouse CD8a Positive Selection Kit (STEMCELL, Singapore) according to the manufacturer's protocol. For stimulation with Ab-coated beads, CD8^+^ cells (4×10^5^) were stimulated in triplicate with mouse T-cell-activator CD3/CD28 beads (Dynabeads, Invitrogen, Waltham, MA ) for 72 h with 30 U/ml recombinant mouse IL-2 (Thermo Fisher Scientific, Waltham, MA).

### Cytotoxicity assay

LLC target cells were washed once in RPMI medium and plated at a concentration of 25,000 cells per 0.1 mL in 96-well microplates. Target cells were co-cultured with 750,000 spleen cells that had been depleted of red blood cells, in a final volume of 0.2 mL at 37°C and in an atmosphere of 5% CO2 and air. After a 6-hour incubation, co-cultured cells were washed three times with PBS. For each target, 3 to 6 replicates of the internal references for the 0% viability background and the 100% viability maximal signal were run. The 0% viability reference point was determined by plating target cells in media with a final concentration of 1% SDS. The 100% viability reference point was determined by plating target cells in media without effector cells.

### Quantification of cell viability and luciferase activity

To facilitate the establishment of a bioluminescent Lewis lung carcinoma (LLC) stable cell line, LLC cells were transfected with luciferase virus from the viral vector core. To determine luciferase activity from the established cell line, LLC cells were seeded in triplicate into 96-well plates at different numbers, starting from 10,000 cells per well to 2×10^5^ cells per well. Cells were lysed 6 h after seeding for quantification of luciferase activity. The results showed that there was a high correlation between the level of luciferase activity and the number of cells seeded (data not shown).

To determine the killing effects of CTLs and NK cells, we analyzed the direct lysis of LLC cells *in vitro* using Promega's Luciferase Assay System. Resting CD8+ T cells and NK cells were first isolated from splenocytes of wild type and Rad52^−/−^ C57Bl/6 mice (described in Materials and Methods) using magnetic bead separation. NK cells were either left unstimulated or transferred to culture in RPMI with IL-2 (1000 U/ml) and incubated for three days at 37°C with 5% CO2. CD8^+^ T cells were further stimulated using Thermo Fisher's Dynabeads Mouse T-Activator CD3/CD28 for T Cell Expansion and Activation and cultured in RPMI with recombinant mouse IL-2 (30U/ml) for three days at 37°C. IL-2-stimulated CTLs and NK cells were added separately to wells with LLCs at an E:T ratio of 30:1 and incubated for 6 and 24 h at 37°C. LLC lysates were then obtained and analyzed for luminescence relative light units (RUIs) as described in Materials and Methods. LLCs without added splenocytes were used as a control to account for the maximum RUI to be detected from the plated number of target cells.

All luciferase assays were performed with Luciferase Assay System (Promega, Madison, WI), and were conducted essentially according to the manufacturer's protocol. For measuring cell-associated luciferase activity, the culture medium was removed. 80 ul of 1X lysis reagent was dispensed into each culture well and plates were shaken gently for 20 min for the cells to be completely lysed. Twenty ul of each sample were transferred to a new white chimney well plate (Greiner Bio-One International, Austria) in triplicate measures and mixed with 100 ul of Luciferase Assay Reagent. The light produced was measured on a Tecan Microplate reader (Switzerland) within 1 min, and the relative light units for each sample were determined.

Percent viability was calculated as the mean luminescence of the experimental sample minus background divided by the mean luminescence of the input number of target cells used in the assay minus background. Percent specific lysis is equal to (1 − percent viability) × 100 and is calculated as follows: % specific lysis = [1 − counts per 5 seconds (experimental - minimum signal) / (maximum signal - minimum signal] × 100.

### ELISpot assays

The mouse granzyme B and IFN-y ELISpot reagents were purchased from R&D Systems, and the assays were performed following the manufacturer's instructions. Briefly, 96-well filter plates (Millipore) were pre-coated with capture Ab overnight. Isolated CD8^+^ T cells and NK cells were plated with and without LLC stimulator cells and incubated, 37°C for 4 h. The plates were washed three times, the detection Ab was added, and the plates with Ab incubated overnight at 4°C. The plates were then developed using the ELISpot Blue Color Module (R&D Systems) and read on an ImmunoSpot (Cellular Technology) ELISpot reader.

### Statistical analysis

Data are presented as mean ± SE. All data were analyzed by the 2-tailed Student t test and corrected as needed using the Bonferroni correction. Multiple testing adjustments were performed so that the threshold would be less than the Bonferroni correction using *p* < 0.05 as threshold.
